# Genomic landscape and expression profile of consensus molecular subtype four of colorectal cancer

**DOI:** 10.3389/fimmu.2023.1160052

**Published:** 2023-06-19

**Authors:** Yujie Lu, Dingyi Gu, Chenyi Zhao, Ying Sun, Wenjing Li, Lulu He, Xiaoyan Wang, Zhongyang Kou, Jiang Su, Feng Guo

**Affiliations:** ^1^ Department of Oncology, The Affiliated Suzhou Hospital of Nanjing Medical University, Suzhou Municipal Hospital, Gusu School, Nanjing Medical University, Suzhou, China; ^2^ Department of Clinical Laboratory, The Affiliated Suzhou Hospital of Nanjing Medical University, Suzhou Municipal Hospital, Gusu School, Nanjing Medical University, Suzhou, China; ^3^ Department of General Surgery, The Affiliated Suzhou Hospital of Nanjing Medical University, Suzhou Municipal Hospital, Gusu School, Nanjing Medical University, Suzhou, China

**Keywords:** colorectal cancer, consensus molecular subtype, genomic mutations, gene expression, tumor immune microenvironment

## Abstract

**Background:**

Compared to other subtypes, the CMS4 subtype is associated with lacking of effective treatments and poorer survival rates.

**Methods:**

A total of 24 patients with CRC were included in this study. DNA and RNA sequencing were performed to acquire somatic mutations and gene expression, respectively. MATH was used to quantify intratumoral heterogeneity. PPI and survival analyses were performed to identify hub DEGs. Reactome and KEGG analyses were performed to analyze the pathways of mutated or DEGs. Single-sample gene set enrichment analysis and Xcell were used to categorize the infiltration of immune cells.

**Results:**

The CMS4 patients had a poorer PFS than CMS2/3. *CTNNB1* and *CCNE1* were common mutated genes in the CMS4 subtype, which were enriched in Wnt and cell cycle signaling pathways, respectively. The MATH score of CMS4 subtype was lower. *SLC17A6* was a hub DEG. M2 macrophages were more infiltrated in the tumor microenvironment of CMS4 subtype. The CMS4 subtype tended to have an immunosuppressive microenvironment.

**Conclusion:**

This study suggested new perspectives for exploring therapeutic strategies for the CMS4 subtype CRC.

## 1 Introduction

According to the National Cancer Center of China, colorectal cancer (CRC) has the second highest incidence among all malignant tumors and is the fourth leading cause of cancer-associated mortality (1). CRC can be divided into different subtypes based on different standards. Tumor node metastasis (TNM) classification and Duke’s classification are traditional classification models for CRC, according to infiltration depth of tumor and metastasis (2). TNM classification is applied predominantly to predict the prognosis of CRC patients (3), as well as to guide the choice of therapeutic schedule.

With the development of medical technology, it has entered into the stage of precise diagnosis and treatment. Genetic variation of different molecular, such as *KRAS*, *NRAS*, *BRAF*, *Her2* and MSI-H, has been applied to guide clinical treatment and prognosis. It has been proved that *KRAS*/*NRAS*/*BRAF* wild-type patients have a better prognosis than *KRAS*/*NRAS*/*BRAF* mutated ones. Bazan et al. compared 74 *KRAS* mutated patients with 86 *KRAS* wild-type and found that patients with codon 13 *KRAS* mutation were related to risk of relapse or death independently (4). Schirripa et al. found that compared to all wild-type patients, *RAS* mutation were related to shorter overall survival (5). *KRAS*/*NRAS*/*BRAF* wild-type patients had a better prognosis when treated with monoclonal antibodies to the epithelial growth factor receptor (*EGFR*) and chemotherapy than treated with chemotherapy only. While addition of Cetuximab to standard chemotherapy couldn’t benefit *RAS* mutated patients (6, 7). What’s more, part of patients with *BRAF V600E* mutation can benefit from combination therapy including *EGFR* and *BRAF* inhibitors (8).

The consensus molecular subtype (CMS) is a developed classification model defined by Guinney et al. in 2015 and is determined by transcriptomic analyses (9). Although the CMS system was originally developed to classify early-stage non-metastatic CRC, it was used to classify metastasis CRC (mCRC) patients in recent several clinical trials (10, 11). CMS can be classified into four subtypes according to the transcriptomics of CRC. Immunohistochemistry of five markers, including *ZEB1*, *FRMD6*, *KER*, *CDX2* and *HTR2B*, can also be used to identify CMS1-4 subtypes (12). Expression of *CDX2* is higher in epithelial-like tumor (CMS2/3), while expression of *HTR2B* and *FRMD6* is higher in mesenchymal-like tumor (CMS4). These five markers can be applied to differentiate mesenchymal from epithelial tumor (12). Suggested by the GALGB/SWOG 80405 trial, CMS2 is the most common subtype both in total and left-sided mCRC, while CMS1 is most common in right-sided mCRC (13).

Compared to the other three subtypes, the CMS4 subtype is revealed to possess high somatic copy number alterations, upregulation of genes related to epithelial mesenchymal transformation (EMT), activation of angiogenesis, transforming growth factor β (TGF-β) signaling and matrix remodeling pathways, notable stromal infiltration. In addition, the CMS4 subtype is also reported to show upregulation of integrin-β3, wound-like responses upregulation and a platelet activation signature (14). Importantly, the CMS4 subtype is confirmed to have poorer OS and relapse-free survival, and is associated with a higher risk of recurrence (15).

There are currently no effective therapies for the majority of mCRC patients, especially CMS4 patients. In the AGITG MAX trial, there is no significance in PFS can be found for the addition of bevacizumab to chemotherapy in CMS4 (16). Most mCRC patients with peritoneal metastases belong to the CMS4 subtype and show resistance to oxaliplatin (17). Compared to CMS2/3 patients treated with first-line chemotherapy, CMS4 patients can’t benefit from the combination of bevacizumab with chemotherapy (18). Thus, the CMS4 subtype is generally considered to be related to therapy resistance (19). Few studies have investigated the genetic landscape and its association with CMS4 and few potential mechanism for the phenomena has been reported.

In this study, we investigated the molecular landscape and profiled gene expression in mCRC with CMS4 subtype. *FBXW7* and *CARD11* mutation only occurred in the CMS2/3 subtypes, while *CTNNB1*, *CDH1* and *CCNE1* mutation merely occurred in CMS4. Mutated genes in CMS4 subtypes were enriched in Wnt signalosome, cellular localization, androgen receptor binding and signaling by FGFR1 pathway, etc. Notch pathway was enriched in the CMS2/3 subtype, while Wnt and cell cycle pathway was enriched in the CMS4 subtype. MATH was found significantly lower in the CMS4 subtype than in CMS2/3. We also first identified a PFS-related gene, several immune-related genes and immunologic signature gene in the CMS4 subtype. It was indicated that the CMS4 group had an immunosuppressive microenvironment. The discovery of our study may guide the select of treatment for CMS4 patients and allow more patients benefit from it in the future.

## 2 Methods

### 2.1 Immunohistochemical staining of tumor specimens

Paraffin-embedded specimens were cut into 4 μm thick sections, baked at 65°C for 60 min, and deparaffinized using leicaBondMax (Leica Biosystems, Wetzlar, Germany). Antigen retrieval was performed in BOND Epitope Retrieval Solution 2 (Cat. No. AR9640, pH9.0, Leica) by heating at 100°C for 20 min. Sections were incubated in 3% hydrogen peroxide for 5 min and rinsed with phosphate buffered saline (PBS). Sections were incubated with anti-FRMD6/Willin antibody (ab218209, dilution 1:150, Abcam, Shanghai, China), Anti-5-HT-2B antibody (HPA012867, dilution 1:2000, Merck, Beijing, China), Anti-CDX2 antibody [EPR2764Y] (ab76541, dilution 1:2000, Abcam, Shanghai, China), Anti-ZEB1 antibody [EPR17375] (ab203829, dilution 1:150, Abcam, Shanghai, China), Anti-pan Cytokeratin antibody [AE1/AE3] (ab27988, dilution 1:100, Abcam, Shanghai, China) for 20 min, respectively. Sections were washed by PBS, followed by incubation with primary antibody at 25°C for 10 min, washing by PBS, and incubation with secondary antibody at 25°C for 10 min. Finally, 3,3’-diaminobenzidine tetrahydrochloride (DAB) staining was performed at 25°C for 10 min and incubated by Hematoxylin for 5 min before sealing the sections.

### 2.2 Patients

Clinicopathological data of 24 patients with mCRC were obtained from the Department of Oncology, the Affiliated Suzhou Hospital of Nanjing Medical University. The patients were divided into two groups (G1 (CMS2/3) and G2 (CMS4)) according to an online IHC mini classifier tool (20) after acquiring IHC staining of *FRMD6*, *ZEB1*, *HTR2B*, *CDX2* and *KER* in tumor specimens (**Figure S1**). Meanwhile, the CMS classification was also separately verified by the CMScaller R package (21) based on transcriptome data. The inclusion criteria were as follows: 1) aged between 18 and 80 years, 2) CRC as the only tumor, 3) confirmed by histopathological diagnosis, 4) treated with standard regimens, 5) CMS1 excluded, and 6) detailed clinical pathology information. All specimens were performed for DNA and RNA analyses, and DNA data of 13 specimens was further analyzed. Written informed consent to participate in the study was obtained from the patients. This study was approved by the ethics board of the Affiliated Suzhou Hospital of Nanjing Medical University (approval number: KL901250).

### 2.3 Targeted DNA sequencing and data analysis

Genomic DNA was acquired from formalin-fixed, paraffin-embedded (FFPE) specimens using the Tianquick FFPE DNA Kit (Beijing, China) following the manual guide. The DNA was quantified using a Qubit dsDNA HS assay kit (ThermoFisher Scientific, Waltham, MA, USA). After shearing the genomic DNA into 150-200 bp fragments using a Covaris M220 Focused-ultrasonicator (Covaris, Woburn, MA, USA), the fragmented DNA was used for library generation per the KAPA HTP Library Preparation Kit (KAPA Biosystems, Wilmington, MA, USA). The DNA library was hybridized using a 579-gene panel (Genecast, Wuxi, China) and sequenced I Illumina Novaseq platform (Illumina, San Diego, CA, USA). For somatic mutation calling, raw data were de-multiplexed. After removing low-quality reads, reads were aligned to the hg19 reference genome using BWA MEM and the aligned sequence was indexed using Samtools. Tumor tissues were analyzed using matched blood samples as controls. Somatic mutations analyzed by Varscan2 were defined as follows: 1) in exonic regions; 2) with a depth of ≥ 100× and an allele frequency of ≥ 5%; and 3) with an allele frequency of ≥ 0.2% in the Exome Aggregation Consortium and the Genome Aggregation Database. The calculation of MATH scores was referenced to Rocco et al. (22). Tumor mutation burden (TMB) (mutations/Mb) was calculated using algorithm as reported by Chalmers et al. (23). Nonsynonymous somatic mutations (variant frequencies no less than 5%) at the exonic and splicing regions were quantified. The total number of mutations counted was divided based on the size of the coding region of the targeted panel to calculate the TMB per megabase.

### 2.4 RNA sequencing and data analysis

RNA was acquired from FFPE samples using Rneasy FFPE Kit (Qiagen, Germantown, MA, USA). The RNA quality was assessed on a 2100 Bioanalyzer (Agilent Technologies, Santa Clara, CA, USA). Samples with high quality of RNA (with DV200 ≥ 25%) were used for subsequent experiments. The mRNA libraries were prepared using the NEBNext^®^ Ultra™ RNA Library Prep Kit and they were sequenced on the Illumina NovaSeq platform. Raw reads were processed to remove low quality sequences (de-junction contamination, rRNA removal, etc). For gene expression analysis, clean reads were aligned to the reference human genome (hg19) using HISAT2 25751142 (http://ccb.jhu.edu/software/hisat2/index.shtml). Transcript assembly was performed using StringTie51 (v1.2.3). FeatureCounts (24) was used to estimate the expression level of each gene. Gene expression was determined by HTSeq. The quantification of gene expression was determined by fragments per kilobase per million mapped reads. We used the DESseq2 package (25) in the R software to screen differentially expressed genes between comparisons. Data were normalized by a negative binomial distribution statistical method. The resulting P values were subjected to multiple test corrections according to the Benjamini and Hochberg methods to exclude false positives. Genes, with |log2(fold change)| > 1 and P < 0.05, were defined as differentially expressed genes (DEGs) by DESseq.

### 2.5 Protein network analysis

For protein network analysis, protein-protein interaction (PPI) network data were obtained to retrieve the Interacting Genes (STRING; https://string-db.org/). An interaction score of > 0.4 was set as the threshold. The PPI network was envisioned by Cytoscape, and hub genes were identified by CytoHubba (26).

### 2.6 Tumor immune microenvironment analysis

For tumor immune composition analysis, gene set enrichment analysis (GSEA) was performed using GSEA tools (http://www.broadinstitute.org/gsea). Innate anti-PD-1 resistance (IPRES) data were downloaded from http://software.broadinstitute.org/gsea/msigdb (27). Single-sample gene set enrichment analysis (ssGSEA) and Xcell were used to quantify the infiltration of different types of immune cells.

### 2.7 Statistical analysis

Statistics was conducted by R package (version 4.0, https://cran.r-project.org/), and different groups were analyzed using Fisher’s exact test. Student’s *t*-test and chi-square test were used to analyze clinical characteristics and categorical variables, respectively. Kaplan–Meier curves were used to predict PFS and compared statistically using log-rank test (28, 29). Statistical significance was set at P < 0.05.

## 3 Results

### 3.1 Clinicopathological characteristics

Patients are classified into two groups according to the IHC expression and the transcriptome-based CMS classification, G1 and G2, represents CMS2/3 and CMS4 subtypes, respectively. The clinicopathological characteristics of CRC patients in the G1 and G2 groups are shown in **Table 1**. The median age is 56 years in both groups (P=0.70). 11 males and 4 females are in the G1 group, while 4 males and 5 females in G2. Most patients are adenocarcinomas (87.5%, 21/24) and others are signet ring cell carcinoma (2/24) and cancerization (1/24). All the G2 patients are adenocarcinomas. Ninety percent of the lesions are located on the left side of colon (21/24). The Eastern Cooperative Oncology Group (ECOG) performance status (PS) score of most patients are lower than 2 (87.5%, 21/24). Mutations in the *KRAS*, *NARS* and *BRAF* genes are more common in the G2 group than those in the G1 group (88.9% vs. 40.0%, P=0.02). The median values of tumor mutational burden (TMB) of the G1 group are 5.3, while those of the G2 group are 3.9 (P=0.31). Among all clinicopathological characteristics, only mutation type is statistically different between the two groups.

**Table 1:** Clinicopathological characteristics of the G1 and G2 patients.

Characteristics	G1, CMS2/3 (N=15)	G2, CMS4 (N=9)	*P* value
Age (median, years)	56	56	0.70
Gender			0.16
Male	11	4	
Female	4	5	
Pathology			0.15
Adenocarcinoma	12	9	
Other Signetring cell carcinoma Cancerization	3 2 1	0 0 0	
Primary site			0.15
Right	3	0	
Left	12	9	
ECOG PS			0.54
0	1	2	
1	12	6	
2	2	1	
Mutations			0.02*
KNB mt^#^	6	8	
RAS	4	8	
BRAF	2	0	
KNB wt^##^	9	1	
TMB (median)	5.3	3.9	0.31

^#^KNB mt represents Ras or Braf mutation. ^##^KNB wt represents Ras and Braf wild-types. *P value < 0.05.

**Figure 1:**
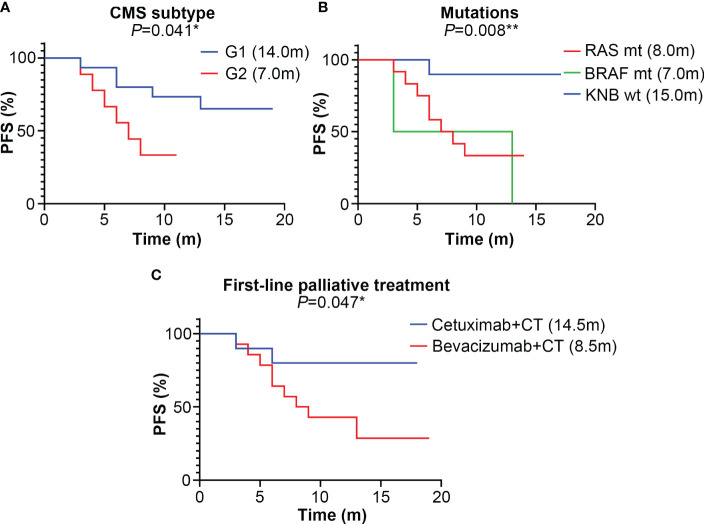
The median progression-free survival (PFS) (month) of different subtype groups of colorectal cancer patients. **(A)** The median PFS of the G1 (CMS2/3) and G2 (CMS4) groups; **(B)** The median PFS of KRAS, NRAS and BRAF mutation subtypes; mt represents mutant-type, wt represents wild-type; **(C)** The median PFS of different first-line palliative treatment subtypes; CT represents chemotherapy. * represents *P*<0.05; ** represents *P*<0.01; *** represents *P*<0.001.

As shown in **Figure 1A**, the PFS of the G2 group (7.0 months) is significantly shorter than that of the G1 group (14.0 months, P=0.041). Compared to patients with *KRAS*, *NRAS* and *BRAF* wild-types (15.0 months), those carrying the *RAS* (8.0 months) or *BRAF* (7.5 months) mutations have shorter PFS (P=0.008, **Figure 1B**). Patients treated with cetuximab and chemotherapy have a significantly longer PFS than those treated with bevacizumab and chemotherapy (P=0.047, **Figure 1C**).

### 3.2 Somatic mutations analyses

**Figure 2:**
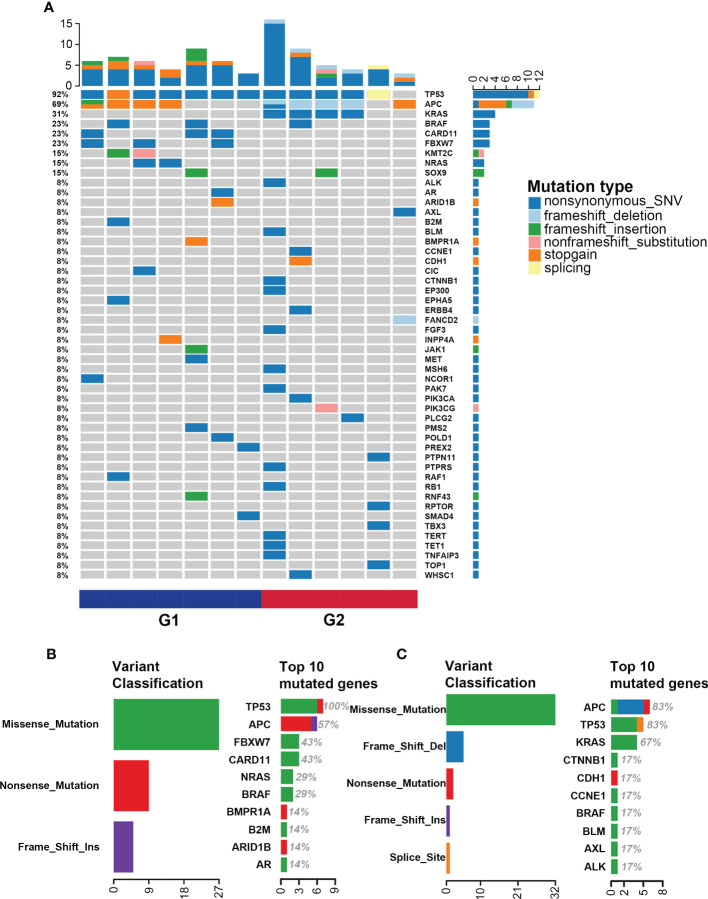
Single nucleotide variation landscape of both groups. **(A)** Landscape of somatic mutations in the G1 (CMS2/3) and G2 (CMS4) groups; **(B)** Detailed information of gene mutations in the G1 group; **(C)** Detailed information of gene mutations in the G2 group.

The landscape of somatic mutations is investigated and the top 50 mutated genes in the G1 and G2 groups are listed in **Figure 2A**. *TP53* (92%), *APC* (69%) and *KRAS* (31%) are the most frequently mutated genes in the whole cohort. Missense mutations, nonsense mutations and frame-shift insertion/deletions are the major types in both G1 and G2 groups (**Figures 2A-C**). Interestingly, with regard to each specific mutated gene, the mutation types are completely different between the two groups. Such as the *APC* gene, nonsense mutation is the major type in the G1 group, while frame-shift deletion is predominant in the G2 group (**Figures 2A-C**). In the G1 group, the top 10 mutated genes are *TP53*, *APC*, *FBXW7*, *CARD11*, *NRAS*, *BRAF*, *BMPR1A*, *B2M*, *ARID1B* and *AR* (**Figure 2B**); while *APC*, *TP53*, *KRAS*, *CTNNB1*, *CDH1*, *CCNE1*, *BRAF*, *BLM*, *AXL* and *ALK* in G2 (**Figure 2C**). Of note, the *FBXW7* and *CARD11* mutations only occur in the G1 group (**Figures 2A, B**), whereas *CTNNB1*, *CDH1* and *CCNE1* mutations predominantly occur in the G2 group (**Figures 2A, C**).

### 3.3 Enrichment analysis of mutated genes in the G1 and G2 groups

Gene ontology (GO) enrichment analysis shows that in the cellular component-associated category, the mutated genes in the G1 group are enriched in HFE-transferrin receptor complex, plasma membrane receptor complex and so on (**Figure S2A**), and the mutated genes in the G2 group are enriched in Wnt signalosome and catenin complex, etc (**Figure S2B**). For biologic process category, the mutated genes in the G1 group are enriched in signal transduction by protein phosphorylation (**Figure S2C**), and those in the G2 group are cellular localization, positive regulation of macromolecule metabolic process and regulation of transferase activity, etc (**Figure S2D**). For molecular function, the mutated genes in the G1 group are enriched in transcription factor activity (**Figure S2E**), and those in the G2 group are androgen receptor binding and kinase binding (**Figure S2F**).

**Figure 3:**
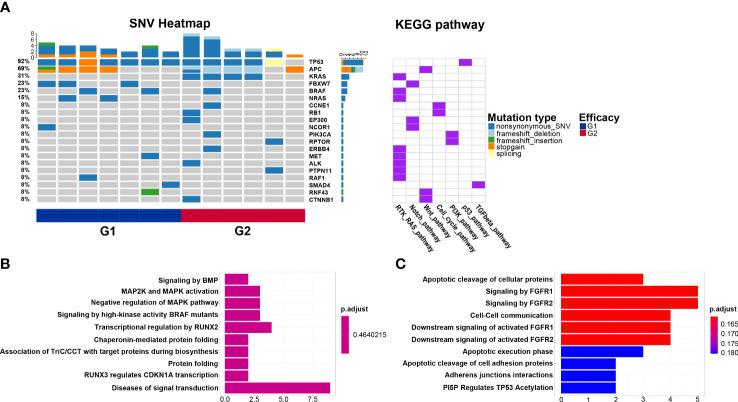
Biological pathways enriched from mutated genes. **(A)** Kyoto Encyclopedia of Genes and Genomes pathways of mutated genes in the G1 (CMS2/3) and G2 (CMS4) groups; **(B)** Reactome pathways of mutated genes in the G1 group; **(C)** Reactome pathways of mutated genes in the G2 group.

KEGG pathway analysis reveals that the Notch pathway, in which the *FBXW7* mutation located, is enriched in the G1 group. The cell cycle pathway that *CCNE1* and *RB1* mutations located in is enriched in the G2 group. Similarly, the Wnt pathway that the *CTNNB1* mutation located in is enriched in the G2 group (**Figure 3A**). Reactome pathway analysis reveals the mutated genes in the G1 group are enriched in transcriptional regulation by RUNX2 pathway (**Figure 3B**), and those in G2 are enriched in signaling by FGFR1 and signaling by FGFR2 pathways (**Figure 3C**).

### 3.4 MATH in the G1 and G2 groups

**Figure 4:**
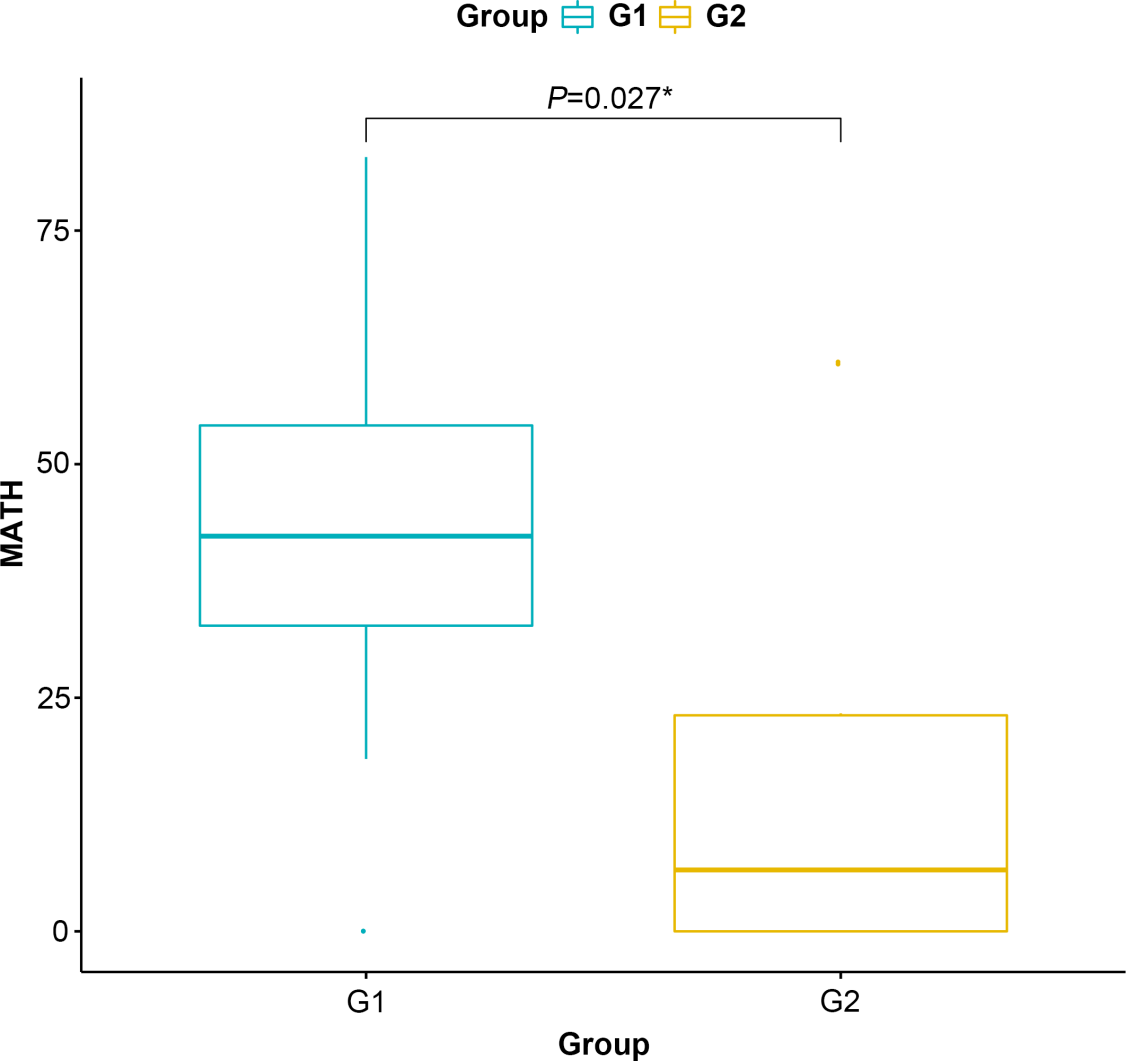
Mutant-allele tumor heterogeneity score of the G1 (CMS2/3) and G2 (CMS4) groups. * represents *P*<0.05.

MATH score is used to quantify intratumor heterogeneity and is predictive for drug resistance and tumor recurrence. Although the TMB value between G1 and G2 groups is insignificant, the MATH score in the G2 group is significantly lower than that in G2 (P=0.027, **Figure 4**), indicating that the level of intratumor genetic heterogeneity of CMS4 patients is lower than that of CMS2/3.

### 3.5 Gene expression profiling in the G1 and G2 groups

**Figure 5:**
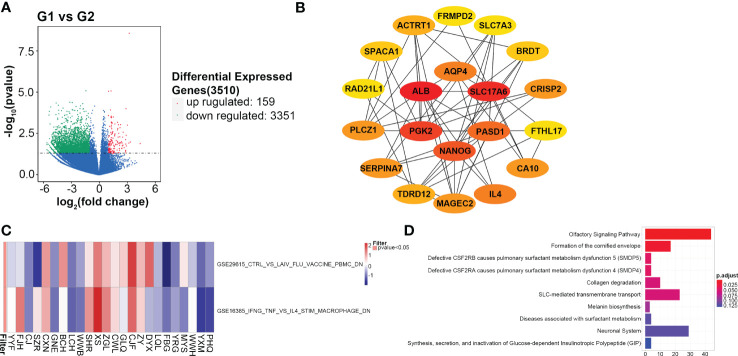
Gene expression profiling analyses. **(A)** Differential expressed genes between the G1 (CMS2/3) and G2 (CMS4) groups; **(B)** Top 20 hub genes in the protein and protein interaction network; **(C)** The enrichment analysis for immunologic signature gene sets; **(D)** Reactome pathways of differential expressed genes.

A total of 3,510 DEGs are identified, and the majority of which are downregulated in the G2 group. The volcano plot of differentially expressed genes is shown in **Figure 5A**. PPI network downloaded from the STRING database is displayed in **Figure S3**. The top 20 hub genes with the highest nodes, including *SLC17A6*, *ALB*, *AQP4*, *PGK2*, *PASD1*, *NANOG*, *FRMPD2*, *SCL7A3*, *BRDT*, *CRISP2*, *FTHL17*, *CA10*, *IL4*, *MAGEC2*, *TDRD12*, *SERPINA7*, *PLCZ1*, *RAD21L1*, *SPACA1* and *ACTRT1*, are shown in **Figure 5B**. Survival analysis of these hub genes shows only *SLC17A6* is associated with the prognosis of CMS4 patients, and higher mRNA expression of *SLC17A6* is associated with worse PFS (P=0.04, **Figure S4**).

Among the differentially expressed genes, at least ten immune-related genes (*CD1C, IDO2, IL4, IL17F, IL1A, CCL3, MAGEC2, KRT5, CEACAM8 and VTCN1*) are found. The expression of these genes is higher in the G2 group than that in G1 except of *CDIC* and *CEACAM8* (**Figure S5**). Of which, *IDO2*, *IL4* and *VTCN1* negatively regulate checkpoint and immune response; *KRT5* is an oncogene that regulate tumorigenesis. *CD1C* stimulates immune response and *CEACAM8* functions as lymphocyte markers (30, 31). Two immunologic signature gene sets, GSE29615 and GSE16395 are identified with high confidence in GSEA (P<0.05, **Figure 5C**). Reactome analysis shows the top ten enrichment pathways, including SLC-mediated transmembrane transport and formation of the cornified envelope (**Figure 5D**).

Thus, through analyzing gene expression profiling in both groups, a PFS-related gene, several immune-related genes and immunologic signature gene sets are first identified in the CMS4 subtype.

### 3.6 Immune-related genes and pathways associated with G2 group

IPRES contains 26 gene signatures that proven to be associated with PD-1 immunotherapy resistance. The IPRES analysis indicates that the immunotherapy resistance of MAPK inhibitor-induced EMT in the G2 group is significantly higher than that in G1 (G1=0.66 vs. G2=0.72, P<0.05). However, other gene sets, such as TGF-β signaling, tumor angiogenesis and VEGFA targets, are not significantly different between the two groups (**Figure S6**).

**Figure 6:**
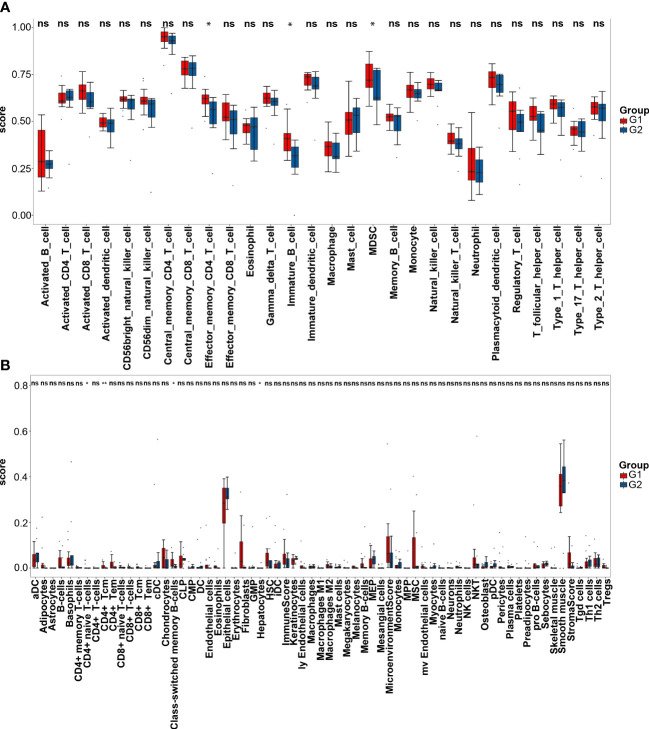
Immune infiltration analyses. **(A)** Single sample gene set enrichment analysis of the G1 (CMS2/3) and G2 (CMS4) groups; **(B)** Xcell analysis of the two groups. * represents P*P*<0.05; ** represents *P*<0.01; ns represents *P*>0.05.

According to ssGSEA analysis, the G2 group is significantly associated with lower infiltration of effector memory CD4+ T cells (P<0.05), immature B cells (P<0.05), and myeloid-derived suppressor cells (MDSC, P<0.05, **Figure 6A**). Xcell analysis shows that some immune cells, including CD4+ T cells, CD8+ T cells, natural killer (NK) cells and macrophages, have no difference in infiltration levels (**Figure 6B**). The infiltration levels of CD4+ naïve T cells (P<0.05), CD4+ central memory T cells (Tcm) (P<0.01) and class-switched memory B cells (P<0.05) are lower in the G2 group, while the level of hepatocytes (P<0.05) is higher in the G2 group. The immune, stroma and microenvironment scores in the G2 group are all lower than those in the G1 group, although there are no statistical differences. Thus, immune-related analyses indicate the CMS4 group has an immunosuppressive microenvironment.

## 4 Discussion

In this study, CMS2/3 or CMS4 subtype was differentiated based on IHC staining with *FRMD6*, *ZEB1*, *HTR2B*, *CDX2* and *KER* markers, which was in line with the transcriptome-based classification system (12, 20). We found that the cell cycle and Wnt pathways were enriched in the CMS4 group. Immunologic signature gene sets and enrichment pathways as well as a novel predictor for CMS4 CRC patients were identified through gene expression analysis. Tumor microenvironment analysis implied a lower immune, stroma, and microenvironment scores in the CMS4 group, which indicated immunotherapy may not be beneficial to these patients. Our results provide a potential mechanism for the poor outcome of mCRC patients with CMS4 subtype and imply different treatment strategies based on the CMS subtype.

In our study, *FBXW7* mutation, the most frequently mutated gene after *TP53* and *APC* in the CMS2/3 group, was not found in the CMS4 group. FBXW7, as a ubiquitin ligase, can combine with lots of cancer-related factors, including c-Myc, cyclin E and mTOR (32–34). *FBXW7* mutation in CRC leads to tumor cell proliferation, increases resistance to paclitaxel, 5-fluorouracil and oxaliplatin, as well as becomes sensitive to mTOR inhibitors (35–37). No correlation between *FBXW7* mutation and CMS4 CRC has been reported in previous studies. *FBXW7* mutation, enriched in the Notch pathway, was not been found in the CMS4 mCRC patients in our study, suggesting that the FBXW7-Notch axis might not be involved in the tumorigenesis of CMS4 CRC. Therefore, treatment targeting Notch or mTOR signaling might not be beneficial to the CMS4 CRC patients.

*CTNNB1* and *CCNE1* were the most frequently mutated genes found in the CMS4 group in our study. *CTNNB1* mutation occurred in about half of CRC patients (38), while the mutation frequency of *CCNE1* in CRC had not been explored. The mutation of these two genes in the CMS4 mCRC had not been reported previously. *CTNNB1* is a significant Wnt signaling regulator that interacts with E-cadherin to mediate cell adhesion (39). The Wnt signaling pathway that *CTNNB1* lies is a critical pathway in EMT, an important feature of CMS4 subtype (20, 40). In our study, *CTNNB1* mutation was enriched in the Wnt pathway, suggesting that *CTNNB1*-Wnt axis might function importantly in the CMS4 mCRC. Further, therapeutic drugs targeting the Wnt pathway, including small molecules, biological agents and natural compounds (41), might be effective treatment of CMS4 subtype mCRC.

*CCNE1* acts as a positive regulator of cell cycle and promotes the transition from G1 to S (42). Abnormal expression of *CCNE1* activates cyclin-dependent kinase 2 to phosphorylate its substrate, resulting in tumor cell proliferation (43). In our study, *CCNE1* mutation was enriched in the cell cycle pathway, suggesting that CCNE1-cell cycle axis might be involved in the tumorigenesis of CMS4 CRC. KEGG pathway analysis also showed that the cell cycle pathway was a unique pathway in the CMS4 type rather than CMS2/3. The arrest of the cell cycle in the G1 phase can be caused by TGF-β, which can induce the cell cycle pathway and effectively inhibit cell proliferation (44). Several studies show that combining ICIs and selective TGF-β inhibitors might be helpful for immunotherapy in CMS4 type mCRC patients (45, 46).

In our study, *SLC17A6*, one member of solute carrier family, was identified as a hub DEG between the two groups, and most hub DEGs were significantly enriched in the SLC-mediated transmembrane transport pathway. Tumor survival, migration, proliferation, and sensitivity to radiotherapy are regulated by *SLC3A2*, and its high expression is associated with poor prognosis (47–49). In a xenograft model, antitumor activity against human colon cancer was mediated by anti-*SLC7A5* monoclonal antibodies (50). The exact roles of *SLC17A6* in CMS4 subtype colon cancer warrant further investigation.

In our study, the mutated genes of the CMS4 subtype were enriched in signaling by *FGFR1* and *FGFR2* pathways according to Reactome pathway analysis. The FGFR tyrosine kinase family regulates migration, differentiation, apoptosis and angiogenesis after ligands (51). A combination of FGFR inhibitors and immune checkpoint blockers is reported to be a promising treatment strategy for malignant tumors (52). However, its application in CMS4 CRC patients requires further research.

According to the GSEA and tumor immune microenvironment analyses, the CMS4 CRC patients tended to have an immunosuppressive microenvironment. MDSCs are a heterogeneous group of cells derived from both myeloid progenitors and immature myeloid cells, which are precursors of dendritic cells, macrophages, and/or granulocytes (53). In our study, fewer MDSCs infiltrated in the CMS4 subtype than in CMS2/43, suggesting that CMS4 CRC cells tended to promote tumor growth (54). GSE16385 is a GEO dataset containing expression data from human macrophages, obtained by comparing macrophages activated by interleukin-4 (M2) and those activated by interferon-gamma and tumor necrosis factor (M1) (55). Macrophages in the immune environment of most cancer cells act as M2 phenotype and express various anti-inflammatory molecules, leading to an immunosuppressive microenvironment (56). Our study found that M2 macrophages infiltrated the tumor microenvironment in most CMS4 samples. Regorafenib transforms tumor-associated macrophage from M2 type to M1 type with anti-tumor function by inhibiting the colony-stimulating factor 1 receptor (57). Meanwhile, regorafenib can inhibit tumor angiogenesis by TIE2 pathway, and reduce proliferation of CMS4 subtype tumor cells in a patient-derived xenograft trail (58, 59). When combined with ICIs, it may have synergistic anti-tumor effect in CRC. One patient with CMS4 in our study, who failed first-line cetuximab and chemotherapy second-line bevacizumab and chemotherapy, was beneficial markedly from the treatment of regorafenib (data not shown). This might change the current clinical practice for mCRC patients with CMS4 subtype, overcome the lack of effective treatment options, and prolong their overall survival. The combination of modalities deserves further studies *in vitro* and *in vivo*. Besides MDSCs and macrophages, there were several other immune cell types infiltrating differently between the two CMS subtypes. Tcm is a long-term T cell derived from naive T cells activated by antigens and can home to lymph nodes to receive antigen re-stimulation. Activated Tcm cells can produce a large number of cloned effective memory T cells carrying the same antigen under the re-stimulation of antigen (60). In our study, the CMS4 subtype tended to have a malignant inflammatory environment that potentially blocked the antitumor effect of active T/immune cells, resulting in a poor immune response.

Additionally, several immune-related genes with significantly different expression levels between CMS4 and CMS2/3 subtypes were identified in our study. IL17F is a member of the IL−17 family of proteins. The investigation by Quan et al. showed that the upregulation of IL17F in mCRC promotes tumor invasion by inducing EMT transition (61) and elevated levels of Th17-associated cytokines in advanced-stage mCRC are associated with poorer overall survival and possible resistance to chemotherapy (62). The high expression of CEACAM8 was reported by Peng et al. (30) to be an independent factor of poor disease-free survival and inversely correlated with CD8+ T lymphocyte cells, predicting distant metastasis and inefficiency of chemotherapy. VTCN1 is an immunoregulatory protein that negatively regulates T cell-mediated immune response in the tumor microenvironment (63). Overexpression of VTCN1 was reported to play an oncogenic role, induce EMT, proliferation, and migration of CRC cells through the Wnt signaling pathway (64) and promote CRC stemness (65). VTCN1 can inhibit T cell activation and proliferation, negatively regulate T cell immune response, and its overexpression promotes tumor tolerance and might contribute to Treg development in a CRC tolerogenic milieu (66). Serving as a negative regulator of T-cell-mediated antitumor immunity, VTCN1 can inhibit T cell activation and cytokine secretion, and regulate cytotoxic T lymphocytes (CTLs) during tumor progression (67).

Our study provides new insights into the molecular characteristic of the CMS4 subtype. The CTNNB1-Wnt and CCNE1-cell cycle axes are likely involved in the tumorigenesis of CMS4 CRC and could be functioned as therapeutic targets. In contrast, the FBXW7-Notch pathway is unlikely involved in the tumorigenesis of CMS4 CRC. The CMS4 CRC patients have been found having an immunosuppressive microenvironment and transforming tumor-associated macrophages from M2 type to M1 type in CMS4 CRC cells might be a therapeutic direction. Through analyzing gene expression profiling in both groups, a PFS-related gene, several immune-related genes and immunologic signature gene sets were first identified in the CMS4 subtype. *SLC17A6*, as a novel predictor for PFS of CMS4 CRC patients, needs further exploration. The study requires more patient recruitment and data collections. Further verification in clinics is warrant.

## Data availability statement

The data presented in the study are deposited in the GSA for Human repository, accession number PRJCA011830.

## Ethics statement

The studies involving human participants were reviewed and approved by The ethics board of the Affiliated Suzhou Hospital of Nanjing Medical University (approval number: KL901250). The ethics committee waived the requirement of written informed consent for participation.

## Author contributions

Contributions: (I) Conception and design: YL, JS, FG. (II) Administrative support: JS, FG. (III) Provision of study materials or patients: YL, DG. (IV) Collection and assembly of data: DG, YS, CZ, WL, LH, XW, ZK. (V) Data analysis and interpretation: YL, DG. (VI) Manuscript writing: All authors. All authors contributed to the article and approved the submitted version.

## Supplementary material

Supplementary Figure 1 Immunohistochemical staining of CMS2/3 and CMS4 subtypes metastasis colorectal cancer.

Supplementary Figure 2 Gene ontology (GO) analysis of mutated genes in the G1 (CMS2/3) and G2 (CMS4) groups. **(A)** Cellular component analysis of mutated genes in the G1 group; **(B)** Cellular component analysis of mutated genes in the G2 group; **(C)** Biological process analysis of mutated genes in the G1 group; **(D)** Biological process analysis of mutated genes in the G2 group; **(E)** Molecular Function analysis of mutated genes in the G1 group; **(F)** Molecular Function analysis of mutated genes in the G2 group.

Supplementary Figure 3 Protein and protein interaction network of the differential expressed genes.

Supplementary Figure 4 Survival curves of patients with different SLC17A6 expression.

Supplementary Figure 5 Average expression of different immune-related genes in the G1 (CMS2/3) and G2 (CMS4) groups.

Supplementary Figure 6 The innate anti–PD-1 resistance analysis of immune-related genes/pathways in the G1 (CMS2/3) and G2 (CMS4) groups.
