# Supplementary figures and images for: Genomic landscape and expression profile of consensus molecular subtype four of colorectal cancer
Source: Front Immunol. 2023 Jun 19;14:1160052. doi: 10.3389/fimmu.2023.1160052 (PMC10315486; doi:10.3389/fimmu.2023.1160052)

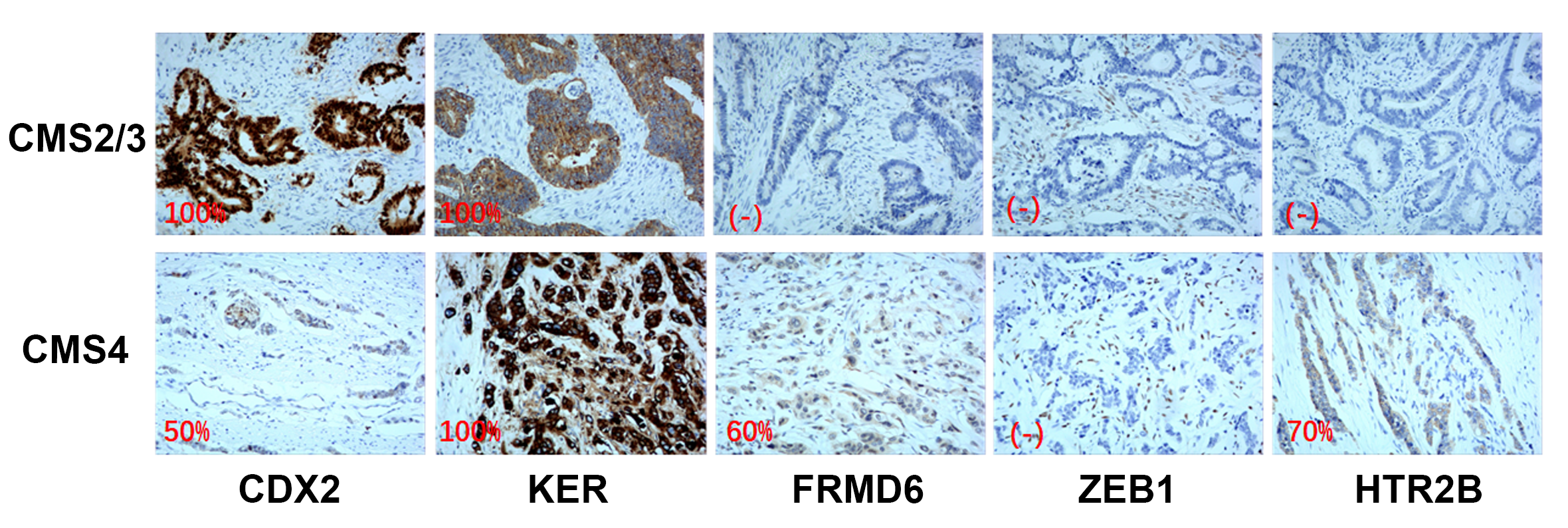

Supplement: Supplementary Figure 1 — Immunohistochemical staining of CMS2/3 and CMS4 subtypes metastasis colorectal cancer. [file DataSheet_1.zip › supplement/S1.tif]

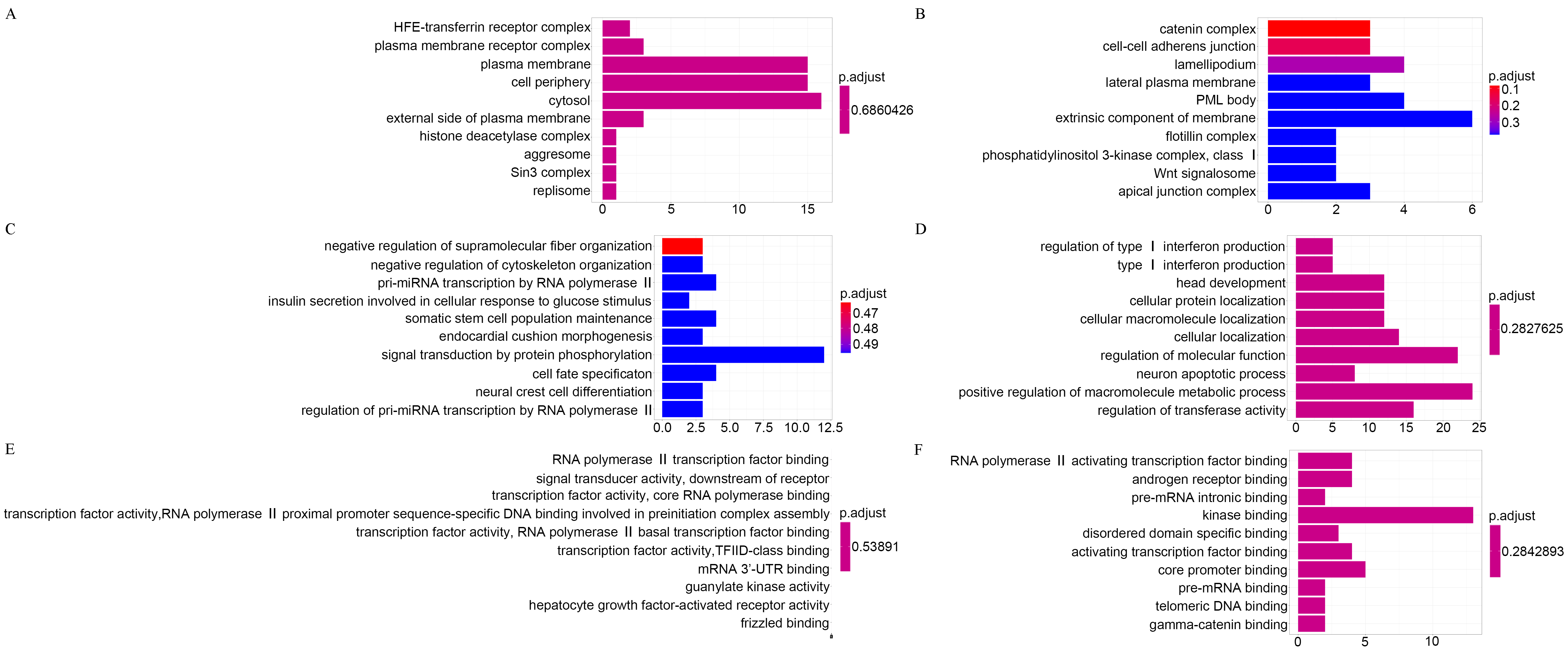

Supplement: Supplementary Figure 1 — Immunohistochemical staining of CMS2/3 and CMS4 subtypes metastasis colorectal cancer. [file DataSheet_1.zip › supplement/S2.tif]

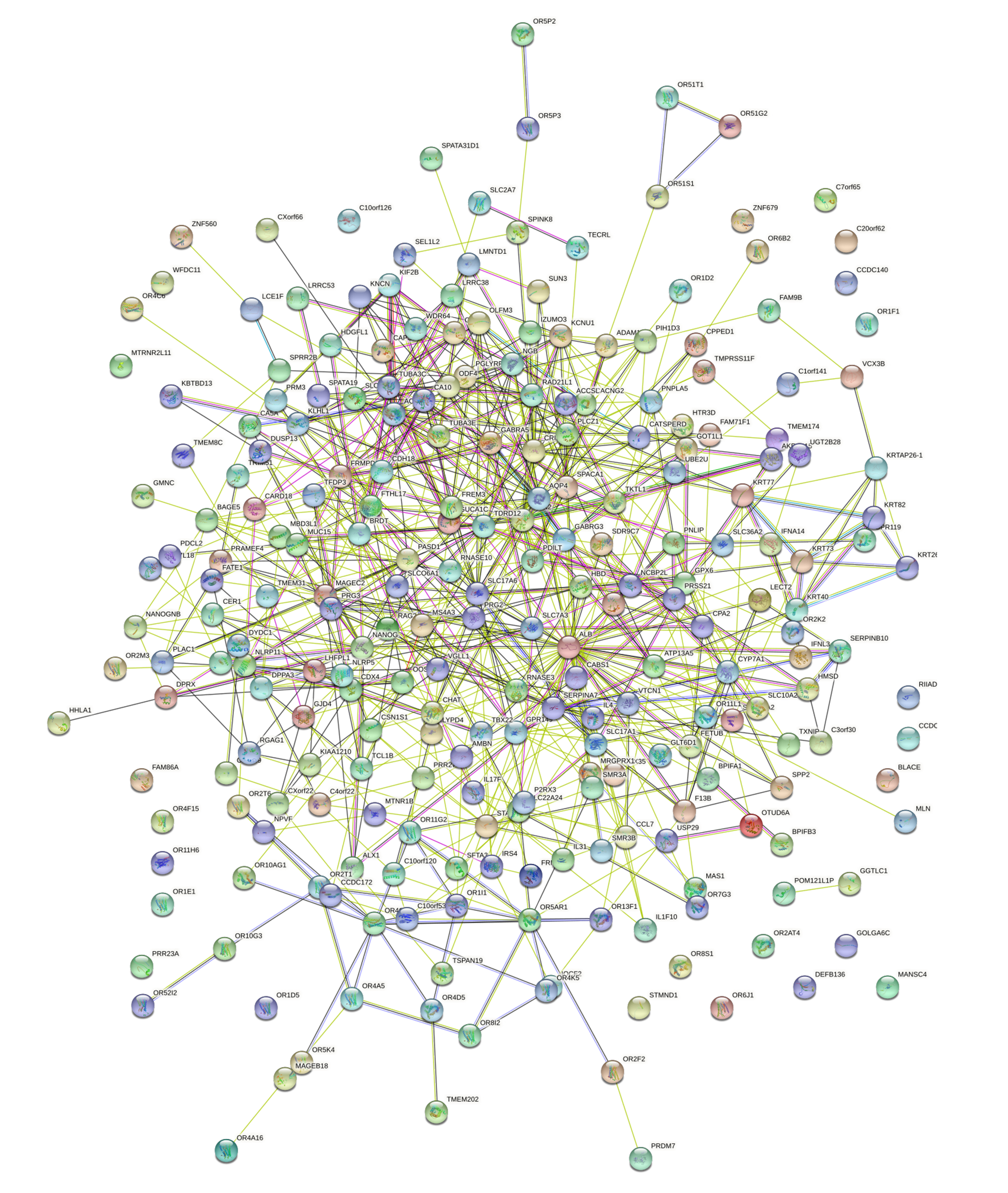

Supplement: Supplementary Figure 1 — Immunohistochemical staining of CMS2/3 and CMS4 subtypes metastasis colorectal cancer. [file DataSheet_1.zip › supplement/S3.tif]

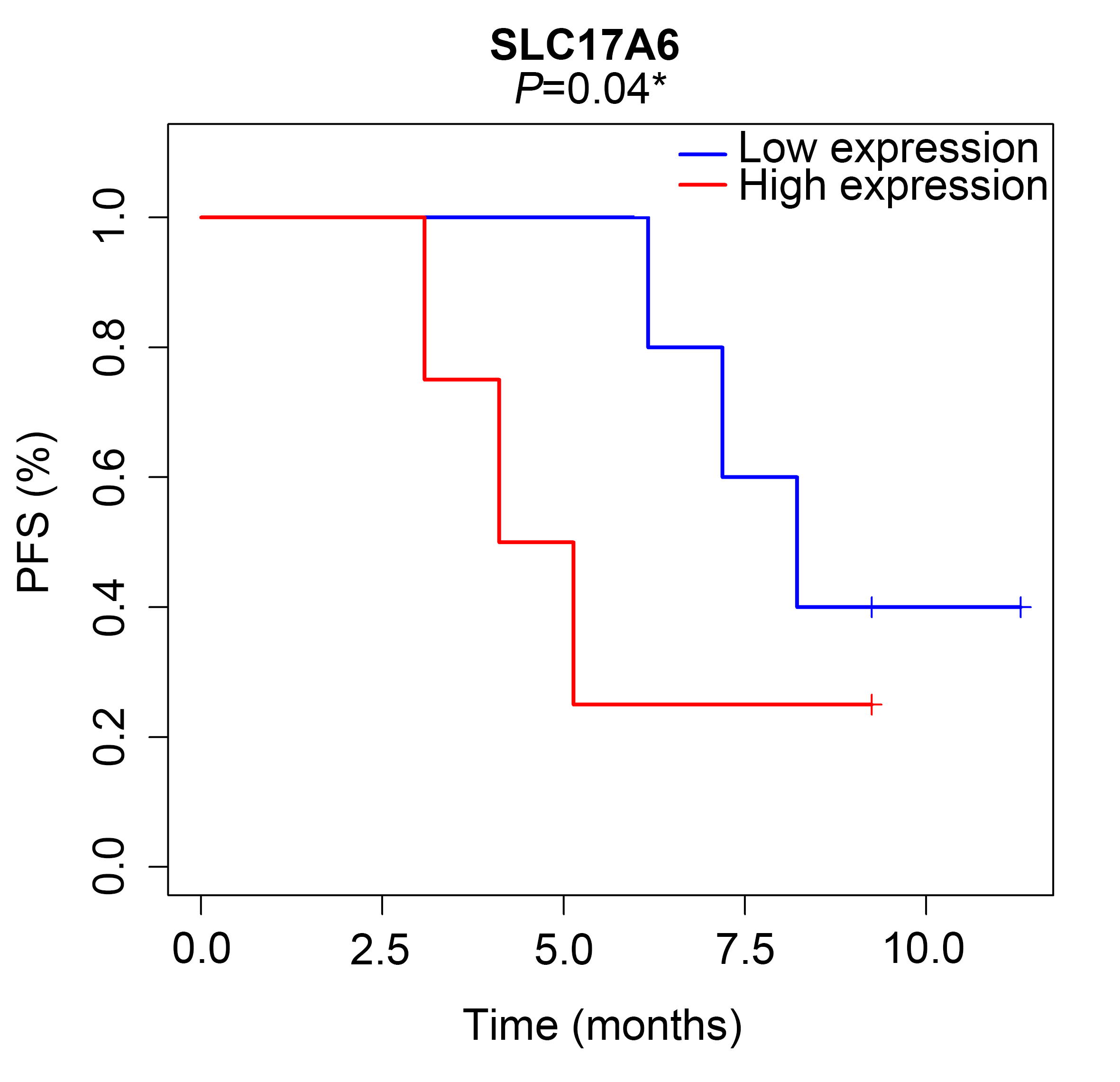

Supplement: Supplementary Figure 1 — Immunohistochemical staining of CMS2/3 and CMS4 subtypes metastasis colorectal cancer. [file DataSheet_1.zip › supplement/S4.tif]

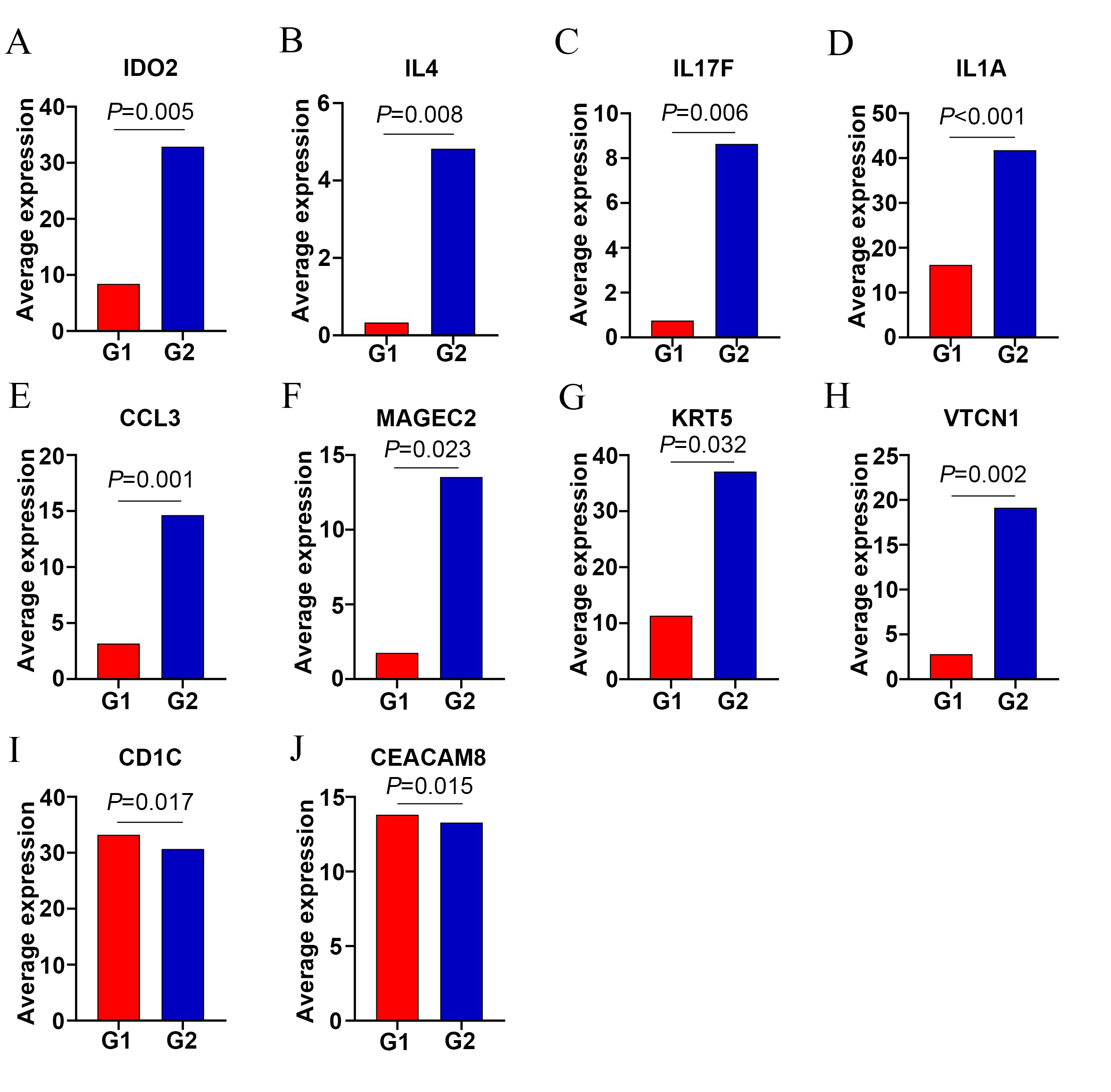

Supplement: Supplementary Figure 1 — Immunohistochemical staining of CMS2/3 and CMS4 subtypes metastasis colorectal cancer. [file DataSheet_1.zip › supplement/S5.tif]

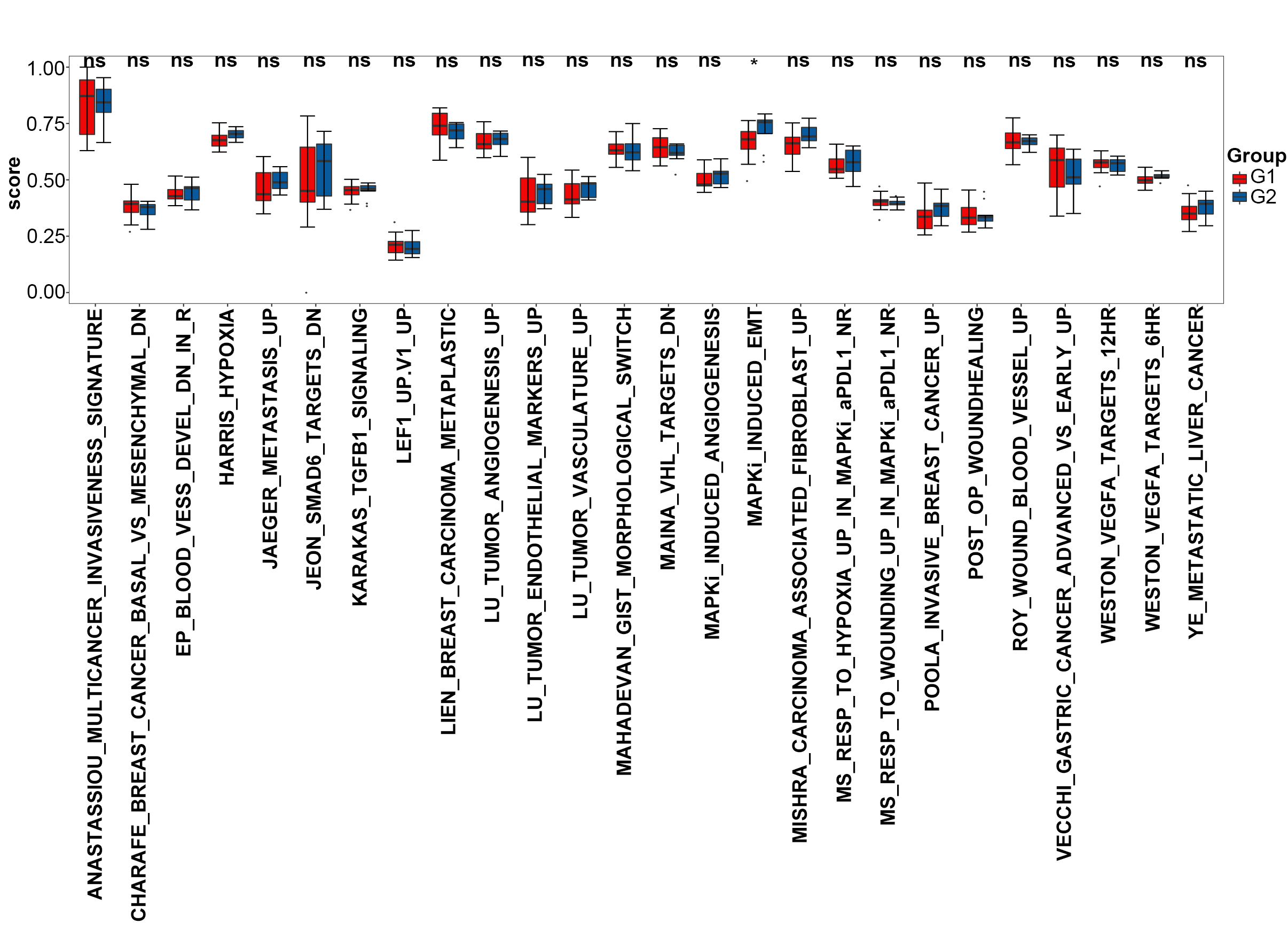

Supplement: Supplementary Figure 1 — Immunohistochemical staining of CMS2/3 and CMS4 subtypes metastasis colorectal cancer. [file DataSheet_1.zip › supplement/S6.tif]
